# The Not-so-Sterile Womb: Evidence That the Human Fetus Is Exposed to Bacteria Prior to Birth

**DOI:** 10.3389/fmicb.2019.01124

**Published:** 2019-06-04

**Authors:** Lisa F. Stinson, Mary C. Boyce, Matthew S. Payne, Jeffrey A. Keelan

**Affiliations:** ^1^Division of Obstetrics and Gynaecology, Faculty of Health and Medical Sciences, The University of Western Australia, Perth, WA, Australia; ^2^Centre for Integrative Metabolomics and Computational Biology, School of Science, Edith Cowan University, Perth, WA, Australia

**Keywords:** meconium, amniotic fluid, PacBio SMRT sequencing, short chain fatty acid, microbiome analysis, fetal microbiome, sterile womb hypothesis, *in utero* colonization

## Abstract

The human microbiome includes trillions of bacteria, many of which play a vital role in host physiology. Numerous studies have now detected bacterial DNA in first-pass meconium and amniotic fluid samples, suggesting that the human microbiome may commence *in utero*. However, these data have remained contentious due to underlying contamination issues. Here, we have used a previously described method for reducing contamination in microbiome workflows to determine if there is a fetal bacterial microbiome beyond the level of background contamination. We recruited 50 women undergoing non-emergency cesarean section deliveries with no evidence of intra-uterine infection and collected first-pass meconium and amniotic fluid samples. Full-length 16S rRNA gene sequencing was performed using PacBio SMRT cell technology, to allow high resolution profiling of the fetal gut and amniotic fluid bacterial microbiomes. Levels of inflammatory cytokines were measured in amniotic fluid, and levels of immunomodulatory short chain fatty acids (SCFAs) were quantified in meconium. All meconium samples and most amniotic fluid samples (36/43) contained bacterial DNA. The meconium microbiome was dominated by reads that mapped to *Pelomonas puraquae*. Aside from this species, the meconium microbiome was remarkably heterogeneous between patients. The amniotic fluid microbiome was more diverse and contained mainly reads that mapped to typical skin commensals, including *Propionibacterium acnes* and *Staphylococcus* spp. All meconium samples contained acetate and propionate, at ratios similar to those previously reported in infants. *P. puraquae* reads were inversely correlated with meconium propionate levels. Amniotic fluid cytokine levels were associated with the amniotic fluid microbiome. Our results demonstrate that bacterial DNA and SCFAs are present *in utero*, and have the potential to influence the developing fetal immune system.

## Introduction

It has long been assumed that the human fetus is sterile. Over the last decade new data have emerged to challenge this dogma. However, these ideas are still controversial and there is disagreement amongst perinatal and microbiological researchers as to whether the human microbiome is seeded prior to birth. While numerous studies have reported the detection of bacterial (Mshvildadze et al., 2010; Gosalbes et al., 2013; Hu et al., 2013; Moles et al., 2013; Ardissone et al., 2014; Hansen et al., 2015; Chu et al., 2016, 2017; Collado et al., 2016; Nagpal et al., 2016; Urushiyama et al., 2017; Wampach et al., 2017; Lim et al., 2018; Shi et al., 2018; Wang et al., 2018), archaeal (Wampach et al., 2017), fungal (Wampach et al., 2017), and viral (Lim et al., 2018) DNA in meconium and amniotic fluid, the interpretation of these data is contentious due to underlying contamination issues.

It is now well accepted that laboratory reagents, including nucleic acid extraction kits and PCR master mix reagents, harbor lows levels of bacterial DNA (Salter et al., 2014). While this contamination is not a major issue for studies of highly colonized samples such as adult feces, it becomes an important issue when working with low biomass samples such as the meconium and amniotic fluid. For example, the presence of bacteria and bacterial DNA in the placenta has previously been reported using 16S rRNA gene sequencing, whole genome sequencing, culture, and staining/microscopy (Stout et al., 2013; Aagaard et al., 2014; Cao and Mysorekar, 2014; Zheng et al., 2015; Bassols et al., 2016; Collado et al., 2016; Gomez-Arango et al., 2017; Parnell et al., 2017). However, issues around contamination and interpretation of low-biomass data remain, with several studies finding that the placental “microbiome” is indistinguishable from negative controls (Lauder et al., 2016; Hornef and Penders, 2017; Leiby et al., 2018; Theis et al., 2019). This is a significant consideration as bacteria entering the intra-amniotic space would necessarily pass through the placenta or the extra-placental membranes.

Until recently, this contamination was largely dealt with by sequencing blank extraction and PCR controls and including these in the analysis. However, we have recently reported a method for removing contamination from PCR master mixes (the “mixome”) which, combined with a carefully performed magnetic bead-based extraction, minimizes contamination in our microbiome workflow to the point that it is barely detectable (Stinson et al., 2018). This allows more sensitive and accurate profiling of low biomass samples. Previous studies of the fetal microbiome are plagued with numerous other methodological issues. In particular, many of these studies use short amplicon sequencing, which is frequently unable to give accurate genus or species level identification (Martinez-Porchas et al., 2016). Several of these studies also perform their analyses using QIIME v1, a pipeline that has been shown to give inaccurate results, with one study suggesting that 56–88% of genus names given by QIIME 1 are false positives (Edgar, 2017). Finally, several of these studies have not taken steps to account for the high levels of PCR inhibitors in meconium, which has led some investigators to conclude that meconium is sterile.

While studies in this field are complicated by methodological problems, those which have at the very least used DNA extraction and amplification controls in their sequencing analyses provide compelling evidence that the intra-amniotic space is unlikely to be sterile in healthy human pregnancies. In fact, transfer of microbes to the developing offspring is a universal trend in Kingdom Animalia, suggesting that there may be an evolutionary advantage to the practice (Funkhouser and Bordenstein, 2013).

Prenatal seeding of the human microbiome would be expected to have significant physiological implications for the developing fetus. The human microbiota produce important metabolites, including short chain fatty acids (SCFAs), which are absorbed systemically and which modify immune function and development (Koh et al., 2016). The presence of bacteria in the intra-amniotic space might trigger immune activation or sensitization, resulting in the production of inflammatory mediators and other immune modulators. Maternal-fetal transmission of microbes during gestation would likely have a significant impact on the fetal immune system, gut, and brain. Additionally, this early scaffolding of the fetal microbiome could influence postnatal colonization events. Thus, it is essential to conclusively determine whether the fetus harbors a microbiome, or whether the purported fetal microbiome is merely the result of contamination and other methodological errors.

The aims of this study were to characterize the bacterial DNA profiles of the fetal gut and amniotic fluid using full-length 16S rRNA gene sequencing and a previously described protocol for minimizing reagent-based contamination (Stinson et al., 2018), taking steps to eliminate environmental contamination as far as possible. Additionally, we profiled levels of inflammatory cytokines in the amniotic fluid and SCFAs in meconium to explore any potential metabolic-immunological-microbiological interactions.

## Materials and Methods

### Patient Recruitment and Sample Collection

Patients giving birth by non-emergency cesarean section between 34 and 42 weeks gestation (*n* = 50) at King Edward Memorial Hospital, Subiaco, Western Australia, were invited to participate in this study with the approval of the Human Research Ethics Committee of the Western Australian Department of Health’s Women and Newborns Health Service (2015212EW). Written informed consent was obtained from all participants. Inclusion criteria were: singleton pregnancies, non-emergency cesarean section deliveries, and a gestational age of ≥34^+0^ weeks. Indications for cesarean section delivery are presented in Table 1. Participants answered a detailed questionnaire regarding their health, diet and lifestyle during their current pregnancy. Clinical data were also collected, including gestational age, maternal age, parity, infant sex, indications for cesarean section, previous obstetric history, and length of stay in neonatal intensive care unit (if any). Maternal and fetal characteristics are reported in Table 2. Five deliveries occurred at late preterm (34–37 weeks gestation). Amniotic fluid samples (*n* = 43) were collected aseptically during cesarean section deliveries. Approximately 10 mL of fluid was drawn into a syringe via a 10 cm cannula fully inserted into the incision site immediately following amniotomy, then transferred to sterile tubes and centrifuged at 40,000×*g* for 6 min at 4°C to pellet cells. Pellets were resuspended in 200 μL nuclease-free water (Integrated DNA Technologies) and both pellets and supernatants were frozen at -80°C until processing.

**Table 1:** Indication for cesarean delivery in this cohort (*n* = 50).

Indication for cesarean section	Number of cases
Previous cesarean section	35
Maternal diabetes	12
Elevated maternal BMI	5
Intra-uterine growth restriction	3
Tubal ligation	3
Tokophobia or previous traumatic vaginal delivery	3
Advance maternal age	3
Macrosomic fetus	2
Breech position	2
Placenta previa	2
Maternal congenital heart defect	1
Placenta accreta	1
Previous myomectomy	1
Colitis	1


*Multiple indications for cesarean delivery were common.*

**Table 2:** Maternal, fetal, and pregnancy characteristics for this cohort (*n* = 50).

Characteristic	Mean (range) or *n* (%)
Maternal age (y)	32.7 (20–44)
Gravidity	3.0 (1–11)
Parity	1.6 (0–7)
GA at birth (week^day^)	38^+2^ (34^+0^–42^+0^)
Pre-pregnancy BMI	29.4 (18.0–52.1)
Smoking in the 3 months prior to pregnancy	4 (8%)
Smoking during pregnancy	3 (6%)
T2 diabetes	6 (12%)
GDM	13 (26%)
Maternal history of asthma	11 (22%)
Maternal history of eczema/dermatitis	6 (12%)
Maternal history of allergies	7 (14%)
Male fetus	26 (52%)


*Values are reported as mean (range) or n (percent).*

First-pass meconium samples (*n* = 50) were collected within the first 24 h of delivery (mean: 8.26 h; range: 0.67–18.55 h). In cases where neonates were fed prior to producing their first meconium, they had received colostrum only. Nappies were removed by gloved midwives and placed in sterile transport bags. Samples were then taken from the nappies in a laminar flow cabinet using aseptic techniques. A central portion of meconium was drawn into a sterile single use syringe to avoid contamination from external portions which may have contacted the neonate’s skin or nappy. Meconium samples were divided into 200 ± 3 mg aliquots and stored at -80°C until extraction.

### Short Chain Fatty Acid Profiling of Meconium

Meconium aliquots (200 mg) from 47 participants (insufficient sample volume in three cases) were transferred to 2 mL Eppendorf tubes. An acidified (0.05 M) 10% aqueous methanol extraction solution spiked with 2-ethyl butyric acid as the internal standard (0.84 mM) was added and the tube was vortexed for 1 min. The tube was left to stand for 60 min at 4°C and then vortexed again for 1 min. The samples were centrifuged at 400×*g* for 20 min at 4°C. Supernatant (1 mL) was transferred to a GC vial and analyzed by GC-MS within 48 h. A blank control was prepared in the same way. Separation of SCFAs was achieved on a Thermo Fisher Scientific GC-MS (ISQ) using a Thermo Fisher Scientific TG-Wax column (30 m × 0.25 mm × 0.25 μm). The injector temperature was set at 230°C in splitless mode and an injection volume of 1 μL. The column temperature was initially 50°C for 0.5 min and then ramped to 110°C over 0.6 min. The temperature was then increased to 135°C at 4°C/min and then ramped to 230°C at 20°C/min and held for 5 min. The MS transfer line was maintained at 230°C and the ion source at 250°C. The MS was operated in electron impact mode and the mass range scanned was 50–250 amu. Quantification was based on a seven-point calibration curve. Acetic acid standards were prepared in the range 0.4–20 mM, butyric and propionic acid in the range 0.2–10 mM, isobutyric acid in the range 0.05–2 mM, isovaleric and valeric acid in the range 0.07–3.6 mM, and isohexanoic and hexanoic acid in the range 0.06–3 mM.

### Cytokine Analysis of Amniotic Fluid

Levels of interleukin 6 (IL-6), interleukin 10 (IL-10), C-X-C motif chemokine 10 (CXCL10), and granulocyte-colony stimulating factor (G-CSF) were measured in amniotic fluid supernatants by multiplex assay (Merck Millipore) on a MAGPIX instrument (Luminex Corp) as per the manufacturer’s instructions. The limits of detection for IL-6, IL-10, CXCL10, and G-CSF were 0.9, 1.1, 8.6, and 1.8 pg/mL, respectively.

### DNA Extraction

DNA was extracted from meconium (*n* = 43) and amniotic fluid pellets (*n* = 43) using a QIAGEN MagAttract DNA/RNA Microbiome kit (QIAGEN Pty Ltd., Hilden, Germany) on the King Fisher DUO platform (Thermo Fisher Scientific) as per manufacturer’s instructions for DNA, with the exception of the bead-beating step, which was performed in 2 mL tubes on a Precellys 24 bead beater at 6,500 RPM for 45 s. Seven out of the 50 collected meconium samples could not be extracted due to adverse reactions with an optional lysis component of the protocol (addition of phenol:chloroform:isoamyl alcohol, 25:24:1 to Solution MBL). This optional step was omitted from all other extractions. One blank extraction control was included in each batch of extractions (11 samples and one control per batch).

### Amplification and Barcoding

PCR was performed on DNA extracted from meconium and amniotic fluid samples to amplify the full 16S rRNA gene for sequencing. The primers 27F (5′-AGRGTTYGATYMTGGCTCAG-3′) and 1492R (5′-RGYTACCTTGTTACGACTT-3′) were used [previously described here (Singer et al., 2016)], with the universal sequences UNITAG-F (gcagtcgaacatgtagctgactcaggtcac) and UNITAG-R (tggatcacttgtgcaagcatcacatcgtag) incorporated at the 5′ end of these. An amine block (5′NH_4_-C_6_) was then incorporated at the 5′ ends of each tagged primer to ensure that unbarcoded amplicons carried over from the first round of PCR did not undergo DNA ligation with SMRTbell adaptors during library preparation. A set of three forward and 15 reverse barcoded primers consisting of a unique barcode sequence [Pacific Biosciences (PacBio)] incorporated with either the UNITAG-F or -R sequence were designed to generate PacBio sequencing-ready amplicons using an asymmetric barcoding strategy [similar to that of Pootakham et al. (2017)]. All primers were synthesized and HPLC-purified by Integrated DNA Technologies.

To obtain barcoded 16S rRNA gene amplicons, amplification was carried out in two steps. The first PCR was carried out in 50 μL reactions containing 7.5 μL of template or water (negative template control), 1X AccuStart II ToughMix (Quantabio), 0.3 μM each of the forward and reverse primers, 1.25 μL each of the ArcticZymes dsDNase and dithiothreitol (DTT) (ArcticZymes), and 13.5 μL of water. PCR mastermix solutions were decontaminated before use using dsDNase as previously described (Stinson et al., 2018). The PCR amplification program consisted of an initial heating step at 94°C for 3 min; 40 cycles of 94°C for 30 s, 55°C for 30 s, and 72°C for 2 min; and a final extension step of 72°C for 7 min. PCR reactions were performed in a Veriti Thermal Cycler (Applied Biosystems). PCR products were visualized on a QIAXcel automated electrophoresis system using a DNA high resolution gel cartridge (run parameters OM500) to confirm the presence and size of amplicons.

Primary PCR products were purified using Agencourt AMPure XP magnetic beads (Beckman Coulter), normalized to 1 ng/μL, then used as template in a secondary, nested PCR, in order to generate asymmetrically barcoded amplicons. Secondary PCR was carried out in 25 μL reactions containing 2 μL of template or water (negative template control), 1X AccuStart II ToughMix (Quantabio), 0.3 μM each of the forward and reverse primers, and 3 μL of water. The PCR amplification program consisted of an initial heating step at 94°C for 3 min; 10 cycles of 94°C for 30 s, 55°C for 30 s, and 72°C for 2 min; and a final extension step of 72°C for 7 min.

### PacBio Sequencing

Barcoded 16S rRNA gene amplicons obtained from the secondary PCR were pooled in equimolar concentrations based on QIAXcel quantitation of the target band (24 samples per pool). Pools were then concentrated using Agencourt AMPure XP magnetic beads and eluted in 50 μL volumes. Pools were next visualized in a 1.2% agarose gel using SYBR Safe DNA stain (Invitrogen) and bands of the appropriate size were excised using a sterile disposable scalpel (new scalpel for each band). Excised bands were purified using a QIAquick Gel Extraction kit (QIAGEN) as per manufacturer’s instructions. 500 ng of purified DNA (amplicon) was used for library preparation of each pool (four pools in total) at the Queensland University of Technology Genomics Research Centre. Here, SMRTbell adapters were ligated onto barcoded PCR products, and the libraries were sequenced on a PacBio Sequel system (version 5.1.0) on a single SMRT cell (per pool). Raw sequence reads have been submitted to the Sequence Read Achieve (project accession number PRJNA530829).

### Sequence Data Analysis

Pacific Bioscience raw reads were processed using the SMRT Link Analysis software (version 6.0) to obtain demultiplexed circular consensus sequence (CCS) reads with a minimum of three full passes and 99.5% sequence accuracy. Sequence data were processed using the software package GHAP v2.1. GHAP is an in-house amplicon processing pipeline developed by Paul Greenfield (CSIRO, Australia) (Greenfield, 2017) built around tools from USearch (Edgar, 2010) and The Ribosomal Database Project (RDP) (Cole et al., 2014), combined with locally written tools for generating OTU tables. BIOM files (McDonald et al., 2012) generated by GHAP were analyzed using MicrobiomeAnalyst – a web-based tool for statistical and visual analysis of microbiome data (Dhariwal et al., 2017). Reads were initially denoized using GHAP with a minimum number of three reads in a minimum of one sample required to retain an OTU. Minimum read filtering was applied in MicrobiomeAnalyst for alpha and beta diversity calculations, but was increased to a minimum of 10% prevalence with a count of 3 for differential abundance analysis. Differential abundance was calculated univariately. All *P*-values were calculated using student’s *t*-test unless stated otherwise.

## Results and Discussion

### Amniotic Fluid and First-Pass Meconium Samples Contain Bacterial DNA

**FIGURE 1:**
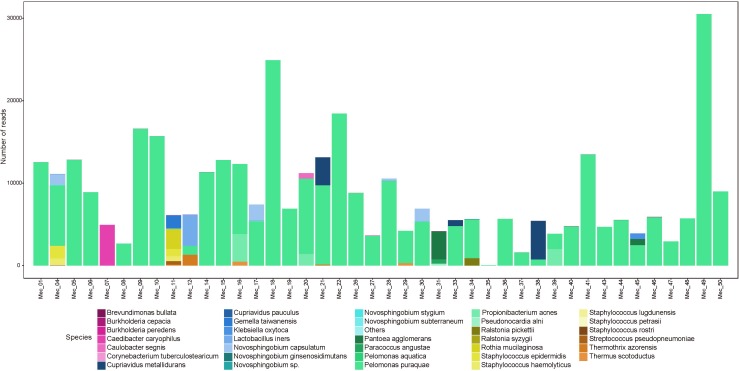
Abundance (number of reads) of bacterial species detected in meconium samples.

First-pass meconium samples were taken as a proxy for fetal gut contents *in utero*. Previous studies have reported the detection of bacterial DNA in meconium samples; however, these data have been disputed due to the possibility of reagent-derived contamination. Here, we have sequenced the full-length 16S rRNA gene in first-pass meconium samples using a protocol that reduced reagent-based contamination to nearly undetectable levels (Stinson et al., 2018). Using these methods, we found all meconium samples to contain bacterial DNA. We recovered an average of 9,081 reads per meconium sample, which mapped to an average of 6.7 OTUs per sample (Supplementary Table S1a). For each polymerase read, we achieved an average of 21 sequencing passes with an average read length of 1555 bp. Almost all meconium samples contained high numbers of reads that mapped to *Pelomonas puraquae* with a 99.5% sequence homology. Five samples did not follow this pattern (Figure 1). One of these was dominated by reads that mapped very poorly to an unknown Rhodospirillaceae, with only 77% sequence homology. Another harbored a relatively high level of diversity and contained mainly reads that mapped to *Staphylococcus* spp. [*S. haemolyticus* and *S. epidermis*, both skin commensals (Cosseau et al., 2016)], *Streptococcus* spp. [including *S. infantis*, an oral and nasopharyngeal commensal (Kawamura et al., 1998) and *S. pseudopneumoniae*], *Gemella taiwanensis* [a newly described species isolated from human blood (Hung et al., 2014)], and *Rothia mucilaginosa* [an oral and upper respiratory tract commensal (Maraki and Papadakis, 2015)]. The third was dominated by reads from *Lactobacillus iners* [a vaginal commensal (Petrova et al., 2017)]. The fourth contained mainly reads from *Pantoea agglomerans* [commonly found in plants, and animal and human feces (Dutkiewicz et al., 2016a,b; Buyukcam et al., 2018)], and the fifth was dominated by reads that mapped to *Cupriavidus gilardii* [previously associated with sepsis (Karafin et al., 2010; Zhang et al., 2017)].

**FIGURE 2:**
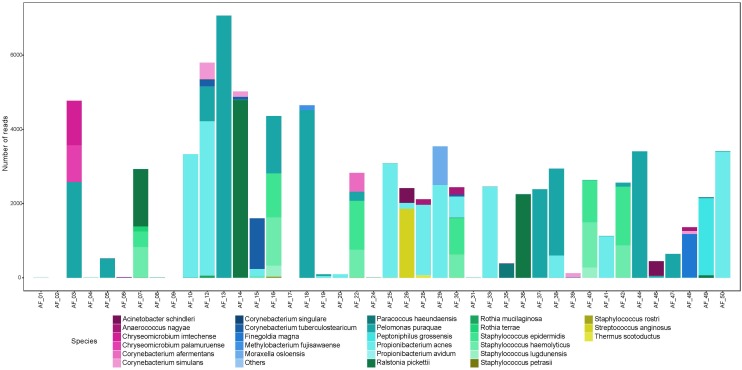
Abundance (number of reads) of bacterial species detected in amniotic fluid samples.

We sequenced an average of 2,196 reads per amniotic fluid sample, which mapped to an average of 7.6 OTUs per sample (Supplementary Table S1b). For each polymerase read, we achieved an average of 24 sequencing passes, with an average read length of 1532 bp. There were seven samples that produced a low number of reads (<50). Overall, amniotic fluid samples contained a low-abundance and low-diversity bacterial microbiome (Figure 2). Unlike meconium, only twelve amniotic fluid samples contained reads that mapped to *P. puraquae*. Other dominant reads included *Propionibacterium acnes*, a human skin commensal, which was also present in twelve samples. Six samples were dominated by reads that mapped to *Staphylococcus* spp. (*S. haemolyticus* and *S. lugdunensis*, both skin commensals). Three samples were dominated by reads that mapped to *Ralstonia pickettii*, a bacterium associated with water and fresh produce, as well as human pathogenic infections and nosocomial infections. One sample was dominated by reads that mapped to *Streptococcus anginosus*, a human commensal that exists in multiple body sites, including the mouth, gut, and vagina. Another was dominated by reads that belonged to *Peptoniphilus grossensis* – a newly discovered bacterium isolated from the feces of a morbidly obese woman. Two of these species, *P. acnes* and *S. lugdunensis*, have previously been cultured from amniotic fluid samples from uncomplicated pregnancies (Collado et al., 2016).

We note that some of the taxa identified here are not biologically plausible human microbiome candidates. In particular, reads mapping to *Thermothrix azorensis* and *Thermus scotoductus*, thermophilic bacteria that are found in extreme environments, were found in very low numbers in two amniotic fluid samples (<100 reads) and in higher numbers in five meconium samples (122–1335 reads). Both of these sequences mapped to these taxa very well (>99% homology); however, in all cases the OTUs mapped just as well to “uncultured bacteria” as they did to these species. To further investigate these sequences we manually trimmed them to either side of the V4 region and classified them using basic local alignment search tool (BLAST) (Altschul et al., 1990). The taxonomic assignments for these OTUs remained as *T. azorensis*, *T. scotoductus*, and “uncharacterized bacteria” using this strategy. These sequences may, therefore, be misclassified, and instead represent currently uncharacterized bacteria which share a high level of sequence homology across the 16S rRNA gene.

### Colonization or Contamination?

Almost all meconium samples were strongly dominated by reads that mapped to *P. puraquae*. One explanation for the appearance of *P. puraquae* in these samples is contamination in our workflow. The ubiquity of *P. puraquae* in our meconium samples might suggest that its appearance is due to a shared source of contamination. Members of the *Pelomonas* genera have previously been identified as contaminants in microbiome reagents (Salter et al., 2014; Glassing et al., 2016) and ultrapure water (Kulakov et al., 2002; Gomila et al., 2005). Indeed, although *P. puraquae* was completely absent in our PCR controls, it did appear in 1/5 of our meconium extraction controls and 2/5 of our amniotic fluid extraction controls (Table 3), so we cannot exclude the possibility of contamination. We have, therefore, presented Figure 1, 2 with the dominant *P. puraquae* OTUs removed in Supplementary Figures S1, S2. However, *P. puraquae* was absent in more than half of our amniotic fluid samples (31/43 samples), and in four of our meconium samples. Thus, its appearance in our extraction controls might be due to sporadic contamination from the samples themselves, as the extraction controls were placed in the center of each extraction plate. The four neonates that lacked *P. puraquae* DNA in their meconium also lacked *P. puraquae* DNA in their amniotic fluid. This adds biological plausibility to the theory that the reads mapping to *P. puraquae* truly were generated from bacterial DNA found in the fetal gut. Another possible source of contamination for these meconium samples is the neonate’s nappy. We did not take swabs of the nappies worn by these infants, but attempted to control for this type of contamination by sampling internalized portions of meconium only. If the *P. puraquae* DNA detected in these meconium samples originated from the nappy, we would not expect to see it in any amniotic fluid samples, which we do. Therefore, we conclude that *P. puraquae*, the primary read recovered from these meconium samples, was unlikely to be purely an external contaminant.

**Table 3:** OTUs detected in blank controls taken during DNA extraction from meconium samples (Meconium extractions EC 1–5) and amniotic fluid samples (AF extractions EC 1–5).

Taxonomic assignment	% match	Meconium extractions					AF extractions				
			
		EC 1	EC 2	EC 3	EC 4	EC 5	EC 1	EC 2	EC 3	EC 4	EC 5
*Pelomonas puraquae*	99.5	8	2	5		6431	42643	600			
*Cloacibacterium rupense*	95.8–98.5		3320								
*Cloacibacterium normanense*	96.8		1142								
Unknown labedella	92.4–94.9		310								
*Pseudoclavibacter soli*	93.3		10								
*Cupriavidus gilardii*	99.1			1476							
*Ralstonia insidiosa*	99.7						4				
*Propionibacterium acnes*	99.9							1			
Unknown rhizobiales	91.9–93.4							28			


*Percent homology to sequences reported on the RDP database is included. All blank PCR controls were completely negative and are therefore not included in this table.*

The majority of reads recovered from our amniotic fluid samples mapped to typical skin commensals, including *P. acnes* and *Staphylococcus* spp. It might be argued that these species are contaminants introduced during sampling or processing. However, it is highly unlikely that skin bacteria could have contaminated these samples during the sampling process. Maternal skin had been excised prior to amniotiomy, and samples were drawn up into a newly opened, sterile syringe directly from within the amniotic sac. While we did not take blank controls during sampling, it is unlikely that these samples were exposed to airborne contamination in the operating theater, as the amount of time that they were exposed to air was negligible. Any user- or reagent-introduced contamination during sample processing would be expected to appear in the extraction and PCR controls that were taken. Apart from a single read that mapped to *P. acnes*, no other skin commensals appeared in these controls (Table 3). Therefore, we conclude that the amniotic fluid contains DNA from human skin commensals that are not representative of contaminants.

Of course, the presence of bacterial DNA in the intra-amniotic space does not prove “colonization.” These bacteria may be transiently transferred from mother to fetus, where they might be unsuited to survive. However, exposure to bacteria and their by-products, even if they do not constitute a true microbiome in the ecological sense, might influence fetal immune development. Alternatively, cell-free DNA from these bacteria may be present in amniotic fluid and meconium samples and thus contribute to the microbial DNA signature. In the present study, however, cell-free DNA is unlikely to be a major contributing factor to the observed bacterial DNA profiles, as DNA is not readily pelleted by centrifugation unless this is done in liquid such as ethanol or isopropanol. Clarification of this question would require confirmation using bacterial culture methods, propidium monoazide, RNA *in situ* probes, or cDNA-based sequencing. While not all previous studies have been able to culture live bacteria from meconium and amniotic fluid samples (Harris and Brown, 1927; Aquino et al., 1984; Escherich, 1989; Rehbinder et al., 2018), many have (Burrage, 1927; Hall and O’Toole, 1934; Snyder, 1936; Jimenez et al., 2008; Moles et al., 2013; Collado et al., 2016). However, it should be noted that in an environment as under-characterized as this, it would be nearly impossible to provide the growth requirements for all bacteria present.

### First-Pass Meconium Contains SCFAs

All meconium samples contained detectable levels of the SCFAs acetic acid and propionic acid, but not butyric acid, iso-butyric acid, valeric acid, iso-valeric acid, hexanoic acid, or iso-hexanoic acid (Table 4). These samples contained an average of 29.35 mmol/g of acetic acid and an average of 4.37 mmol/g of propionic acid. Previous studies have found a similar ratio of acetic acid and propionic acid in infant stool collected on days 2, 3, 5, and 6 of life (Rasmussen et al., 1988; Koenig et al., 2011; Nogacka et al., 2017). These studies were also able to detect very low levels of butyric acid. Given that our own samples were collected on the first day of life, butyric acid-producing bacteria may not colonize the gut until after birth. Our data support previous observations that the SCFA profiles of infant stool differs from that of adult stool, which typically contains equal levels of propionic acid and butyric acid (Hoverstad et al., 1984; Rasmussen et al., 1988).

**Table 4:** Short chain fatty acid levels (mmol/g) in first-pass meconium samples (*n* = 47).

SCFA	Mean (range)
Acetic acid	29.35 (6.32–88.64)
Propionic acid	4.37 (2.78–7.11)
iso-Butyric acid	Not detected
Butyric acid	Not detected
iso-Valeric acid	Not detected
Valeric acid	Not detected
iso-Hexanoic acid	Not detected
Hexanoic acid	Not detected


*Values are reported as mean (range).*

### Is the Presence of *P. puraquae* in These Samples Biologically Plausible?

The most abundant sequence recovered from our meconium samples mapped to *P. puraquae*. Despite our best efforts, this species may represent contamination in our workflow. Here, we consider whether this result is biologically plausible. Importantly, these results differ considerably from those previously reported in the literature. Existing studies have reported that the meconium microbiome is dominated by members of the Enterobacteriaceae family (particularly *Escherichia* spp. and *Enterobacter* spp.), *Propionibacterium* spp., and lactic acid-producing bacteria (including *Lactobacillus* spp., *Leuconostoc* spp., *Enterococcus* spp., and *Lactococcus* spp.) (Gosalbes et al., 2013; Hu et al., 2013; Ardissone et al., 2014; Chu et al., 2016, 2017; Collado et al., 2016; Wampach et al., 2017). *Pelomonas* spp. have never before been reported to be a major part of the meconium microbiome. Additionally, most previous studies report a higher level of within-sample and between-sample diversity. It is unusual to have samples that are so strongly dominated by reads from a single species. One explanation for these discrepancies is the fact that this is the first study of the meconium microbiome to be performed in Australia. Geographic variations in the human microbiome are well established, and Australia, being a relatively isolated country, may be unique in this regard (Moeller and Ochman, 2013; Suzuki and Worobey, 2014; Gupta et al., 2017; He et al., 2018). In fact, *Pelomonas* sp. has previously been identified as a part of the Australian drinking water microbiome (Shaw et al., 2015).

The apparently high level of diversity seen in previous studies of the meconium microbiome might also be due to methodological errors. In particular, several previous studies have used QIIME v1 for OTU clustering and taxonomic assignment. QIIME v1 has recently been shown to overestimate the number of OTUs in a given sample. In fact, up to 88% of predicted genus names given by QIIME v1 may be false positives (Edgar, 2017). Contamination may also account for some of the bacteria that have previously been reported as part of the meconium microbiome. *Escherichia* spp. are often described as a major part of the meconium microbiome; however, we have previously shown that *Escherichia* sp. DNA is a major contaminant of PCR reagents, possibly due to remnant DNA from the *E. coli* used to produce *Taq* DNA polymerase (Stinson et al., 2018). Here, we have decontaminated our PCR reagents using a dsDNase treatment, which we have previously shown to dramatically reduce reagent-based contamination by 99% (Stinson et al., 2018). Further, in studies in which the samples used were not first-pass meconium samples, or in which samples were collected days after birth, sample contamination from the *ex utero* environment may occur. Neonates are rapidly colonized by bacteria from breastmilk and the environment following birth, so it is essential to take only early, first-pass meconium samples to use as proxies of the fetal gut at birth (Perez-Munoz et al., 2017). In the present study, all meconium samples used were passed in the first day of life, an average of 8.26 h following birth. However, previous studies have included samples passed multiple days after birth, casting doubt on their ability to represent the fetal gut contents at birth. Additionally, previous studies of the meconium microbiome have largely relied on short amplicon sequencing, while we have used full-length 16S rRNA gene sequencing. Short amplicon sequencing lacks the taxonomic sensitivity and specificity of full-length amplicon sequencing, and can result in the generation of false positives (Martinez-Porchas et al., 2016). Short amplicon sequencing can be more efficient at producing amplicons in some samples due to DNA fragmentation. On the other hand, while full-length primers generate amplicons that provide superior taxonomic data, they generally have reduced coverage compared to some short amplicon primers. The primers used here cover 81.2% of full-length 16S rRNA gene sequences for the domain Bacteria in the SILVA database, given 1 mismatch within 5 bases of the 3′ end. Based purely on primer coverage, we cannot dismiss the possibility that the results presented here may be an underestimation of the full bacterial microbiome of these samples. Additionally, short PCR amplicons are typically generated more easily than long PCR amplicons.

*Pelomonas puraquae* was highly abundant in these meconium samples. Highly abundant sequences can overwhelm PCR reactions, leading to an under-estimation of diversity. In future work, it may be important to design blocking primers to prevent amplification of *P. puraquae* DNA to allow less abundant sequences to be amplified. This approach has previously been successfully used to detect low abundance species in ticks and krill (Vestheim and Jarman, 2008; Gofton et al., 2015).

We are not the first to identify *Pelomonas* spp. as a colonizer of the naïve human gut. A recent study performed in Crohn’s disease patients tracked microbial assemblage and recolonization following ileocolonic resection and colonic cleansing. These authors found that patients who had recurrence of the disease following surgery had distinctly different recolonization processes than those who were in disease remission. For those patients who experienced disease recurrence, bacterial recolonization followed one of five trajectories. One of these trajectories was affiliated with OTUs belonging to the Comamonas and Pelomonas genera (Mondot et al., 2016). These findings support the notion that *Pelomonas* spp. may be able to colonize naïve human gut environments, and also support other research showing that Pelomonas is a dominant genera in the mucosa-attached microbiota of patients with severe Crohn’s disease (Schaffler et al., 2016). *Pelomonas* spp. have also been identified in human blood (Gyarmati et al., 2015), in the oral microbiome (Mukherjee et al., 2018), and in endometrial samples (Fang et al., 2016; Verstraelen et al., 2016). None of the aforementioned studies, however, controlled for reagent-based contamination, or were able to achieve species level resolution (all short amplicon sequencing studies), so it is difficult to make conclusions on the occurrence of *P. puraquae* in human tissue. Nevertheless, *Pelomonas* spp. DNA has been recovered from non-pregnant endometrial samples, potentially indicating that the uterus is the source of the bacterial DNA that we recovered in meconium and amniotic fluid samples.

### Presence and Abundance of *P. puraquae* in Meconium Is Inversely Correlated With Meconium Propionate Levels

Given the ubiquity of *P. puraquae* in these samples, we divided the meconium samples based on whether *P. puraquae* was absent, present but not dominant, or dominant. For this purpose we defined *P. puraquae* dominance as meaning that the number of reads assigned to *P. puraquae* was greater than the sum of all other reads. This allowed us to compare characteristics based on the presence and abundance of *P. puraquae*. Meconium samples that were dominated by reads belonging to *P. puraquae* had significantly lower concentrations of propionic acid than those that were not dominated by *P. puraquae* (*P* = 0.016). Similarly, *P. puraquae* presence in meconium samples, regardless of whether or not it was dominant, was associated with significantly lower levels of propionic acid in these samples also (*P* = 0.00055). *P. puraquae* is able to grow on acetate (Gomila et al., 2007), the primary SCFA detected in these samples; however, its ability to utilize or produce propionate has not been described.

### Detection of *P. acnes* and *P. puraquae* in Amniotic Fluid Is Associated With Altered Amniotic Fluid Cytokine Profiles and Microbial Richness

**FIGURE 3:**
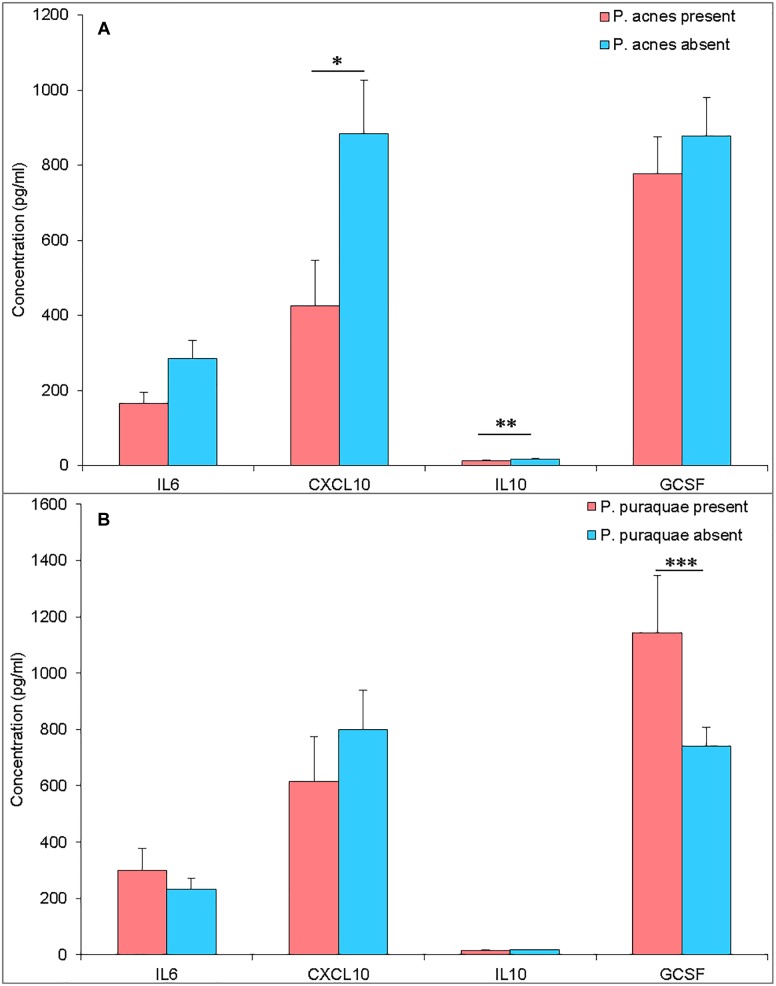
**(A)** Amniotic fluid cytokine levels (pg/mL) from pregnancies with *P. acnes* DNA present (pink) or absent (blue) in the amniotic fluid. **(B)** Amniotic fluid cytokine levels (pg/mL) from pregnancies with *P. puraquae* DNA present (pink) or absent (blue) in the amniotic fluid. ^∗^*P* = 0.058, ^∗∗^*P* = 0.025, ^∗∗∗^*P* = 0.018.

*Propionibacterium acnes* is a ubiquitous human skin commensal. The full genome of *P. acnes* has been sequenced, revealing its potential to produce immunogenic proteins (Bruggemann et al., 2004). Thus, its presence in the amniotic fluid could suggest that fetuses are exposed to bacterial-derived immune stimulation prior to birth. Interestingly, the twelve mothers who harbored *P. acnes* DNA in their amniotic fluid were found to have lower levels of amniotic fluid CXCL10 and IL10 (*P* = 0.058 and 0.025) (Figure 3A). However, *P. acnes* presence within the amniotic fluid was also associated with a significant increase in amniotic fluid microbial richness (number of observed OTUs, *P* = 0.029). The observed reduction in cytokine levels in *P. acnes* colonized amniotic fluid may, therefore, be due to tolerance to a range of bacteria.

*Pelomonas puraquae* DNA was detected in twelve amniotic fluid samples and was the dominant read in eight of these. G-CSF was elevated in amniotic fluid samples that contained *P. puraquae* DNA (*P* = 0.018) (Figure 3B). Amniotic fluid samples in which *P. puraquae* DNA was present, but not dominant, had a significantly higher level of alpha diversity (number of observed OTUs) than samples in which *P. puraquae* was the dominant read (*P* = 0.0021). Given that *P. puraquae* may be an unavoidable contaminant in our workflow, we removed this OTU from our dataset and re-analyzed the relationship between amniotic fluid cytokine levels and bacterial load, alpha diversity, and beta diversity. We were unable to detect any such relationship.

### Meconium Might Be Seeded From the Amniotic Fluid Microbiome

**FIGURE 4:**
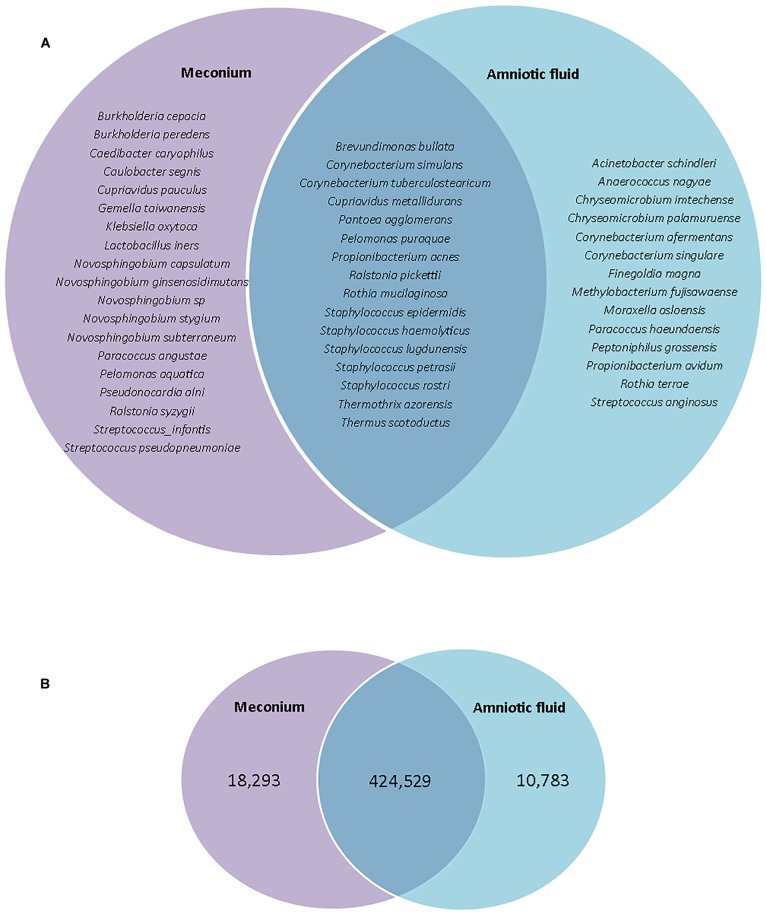
**(A)** Venn diagram showing species which are unique to meconium or amniotic fluid samples, or shared between them. **(B)** Venn diagram showing number of reads that were unique to meconium or amniotic fluid samples, or shared between them.

Given that the fetus swallows amniotic fluid throughout the second and third trimesters, the amniotic fluid and meconium microbiomes may be expected to share a large portion of their microbiota. Indeed, previous studies have shown that the meconium microbiome shares more features with the amniotic fluid microbiome than with the maternal feces, placenta, colostrum, or infant feces (Collado et al., 2016). Here, we found that 32.7% of detected species were shared between amniotic fluid and meconium samples, while 28.6% were found in the amniotic fluid only and 38.8% were found in the meconium only (Figure 4A). However, the 32.7% of shared species accounted for 93.6% of total reads, while only 2.4% of reads were found in the amniotic fluid only and only 4.0% of reads were found in meconium only (Figure 4B). Given that we cannot eliminate the possibility that *P. puraquae* is a contaminant in our workflow, we re-analyzed our OTU table without this species. Without *P. puraquae* 72.6% of reads are shared between the two sample types. Although 16S rRNA gene-based microbiome studies are only semi-quantitative at best, these data indicate that the majority of bacterial DNA found in the fetal gut is also found in the amniotic fluid.

**FIGURE 5:**
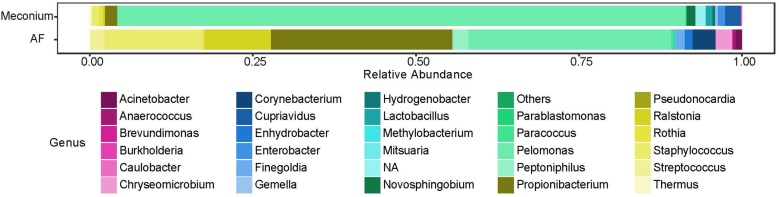
Relative abundance of bacterial genera recovered from meconium and amniotic fluid (AF) samples.

**FIGURE 6:**
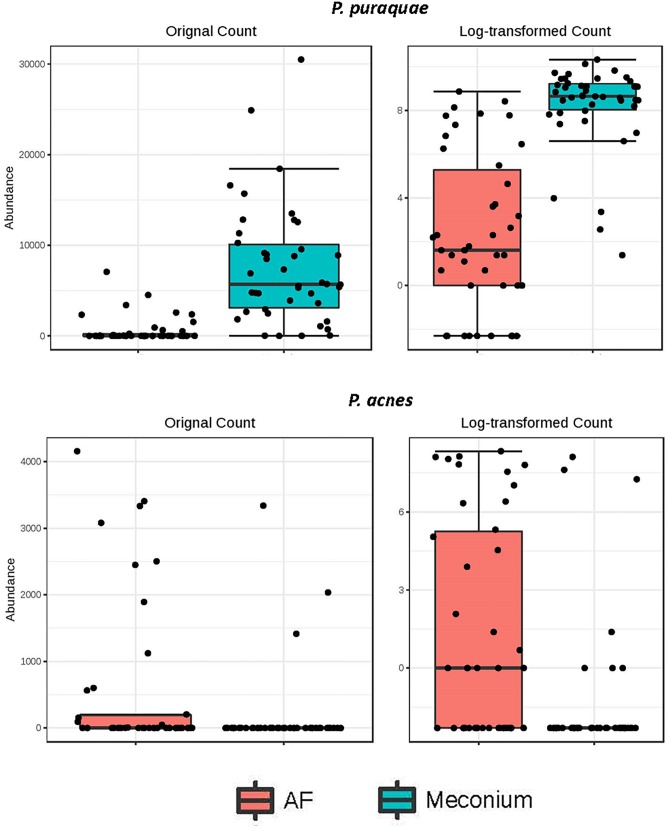
Differential abundance of *P. puraquae* and *P. acnes* in amniotic fluid (pink bars) and meconium (blue bars). Absolute abundance is displayed in the left panels, and log-transformed abundance is displayed in the right panels.

While the majority of reads recovered mapped to species that were shared between the two sample types, there were distinct differences in the relative abundances of these reads (Figure 5). Univariate analysis revealed that *P. puraquae* was significantly more abundant in meconium samples (Mann Whitney *U* test, *P* = 4.39 × 10^-10^), while *P. acnes* was significantly more abundant in amniotic fluid samples (Mann Whitney *U* test, *P* = 0.0078) (Figure 6).

Interestingly, *P. puraquae* was strongly dominant in meconium samples, but was only present in twelve amniotic fluid samples and only dominant in eight of these. Importantly, subjects that lacked *P. puraquae* in their meconium samples also lacked it in their amniotic fluid samples. One hypothesis that may explain these observations is that *P. puraquae* may have a competitive advantage over other bacteria in the fetal gut. Meconium is a very unusual substance, made up of bile acids and salts, pancreatic secretions, epithelial cells, and the residue of swallowed amniotic fluid. *P. puraquae* might be well suited to this environment, while other bacteria might be outcompeted or unable to survive in meconium. Similarly, amniotic fluid samples were largely dominated by skin commensals such as *P. acnes* and *Staphylococcus* spp., which may be suited to such a niche, as amniotic fluid contains high levels of sloughed fetal skin cells (Akiyama and Holbrook, 1994). Thus, these data may demonstrate bacterial niche development *in utero*.

### Meconium and Amniotic Fluid Microbiomes Do Not Vary With Maternal Health Parameters

Aside from the dominant *P. puraquae* sequences, the bacterial DNA profiles of meconium and amniotic fluid in this cohort were remarkably heterogeneous between individuals. We collected a range of maternal health and lifestyle data from the women in our cohort; however, neither beta diversity nor OTU presence/abundance varied with any of these parameters (including maternal pre-pregnancy BMI, parity, gravidity, maternal diabetes, maternal asthma or atopy, maternal smoking, maternal ethnicity, and gestational age at delivery). This was true whether or not *P. puraquae* was removed from the analysis. These results stand in contrast to previous studies which have found that both the amniotic fluid (Wang et al., 2018) and the meconium (Hu et al., 2013) microbiomes vary with maternal diabetes status. Lack of power may be a factor here: our cohort included six mothers with type II diabetes mellitus (T2DM) and thirteen with gestational diabetes (GDM). Although no significant differences in the microbiota were observed in these women, we did find that neonates born from mothers with T2DM had significantly lower levels of propionic acid in their meconium compared to those born from mothers with normal pancreatic function (*P* = 0.0048) or from mothers with GDM (*P* = 0.00328). These differences may be driven by the maternal microbiome. The gut microbiota have been implicated in the pathophysiology of diabetes, with the production of SCFAs suggested as a mechanistic pathway linking microbes and T2DM (Tilg and Moschen, 2014). Thus, mothers with T2DM would be expected to harbor altered gut microbiomes and altered circulating SCFA profiles. These SCFAs might be transferred across the placenta to the fetus.

It has been theorized that fetal microbiome establishment might vary in relation to maternal atopic disease (Koleva et al., 2015). Gosalbes et al. (2013) reported shifts in the meconium microbiota that were related to maternal eczema, but not to asthma or rhinitis. In the present study, we did not find any difference in alpha diversity, beta diversity or community membership based on maternal asthma, allergies, hay fever, or eczema. However, small cohorts such as the one used here lack the power to detect changes in “normal” microbiota (Boers et al., 2016).

## Summary

Here we have provided the first full-length 16S rRNA gene survey of meconium and amniotic fluid. Our data suggest that the fetus is exposed to bacterial DNA and metabolites prior to birth. Additional studies that utilize pre-extraction sample treatment with membrane-impermeable dyes such as propidium monoazide to prevent PCR amplification of DNA contained within compromised cell walls will help to shed light on whether this DNA originates from viable or dead bacteria and as such whether it constitutes a true microbiome.

## Data Availability

The datasets generated for this study can be found in Sequence Read Archive, PRJNA530829.

## Author Contributions

LS recruited all patients involved in the study and collected all samples. MB developed the SCFA analysis. LS and MB performed this experiment. LS performed all other experiments and all data analysis and wrote the manuscript. MB, MP, and JK edited the manuscript. All authors contributed to study design.

## Conflict of Interest Statement

The authors declare that the research was conducted in the absence of any commercial or financial relationships that could be construed as a potential conflict of interest.

## Supplementary Material

Figure S1 Abundance (number of reads) of bacterial species detected in meconium samples presented with the two dominant *P. puraquae* OTUs removed.

Figure S2 Abundance (number of reads) of bacterial species detected in amniotic fluid samples presented with the two dominant *P. puraquae* OTUs removed.

Table S1 **(A)** Meconium OTU table. **(B)** Amniotic fluid OTU table.
