# Supplementary figures and images for: The Not-so-Sterile Womb: Evidence That the Human Fetus Is Exposed to Bacteria Prior to Birth
Source: Front Microbiol. 2019 Jun 4;10:1124. doi: 10.3389/fmicb.2019.01124 (PMC6558212; doi:10.3389/fmicb.2019.01124)

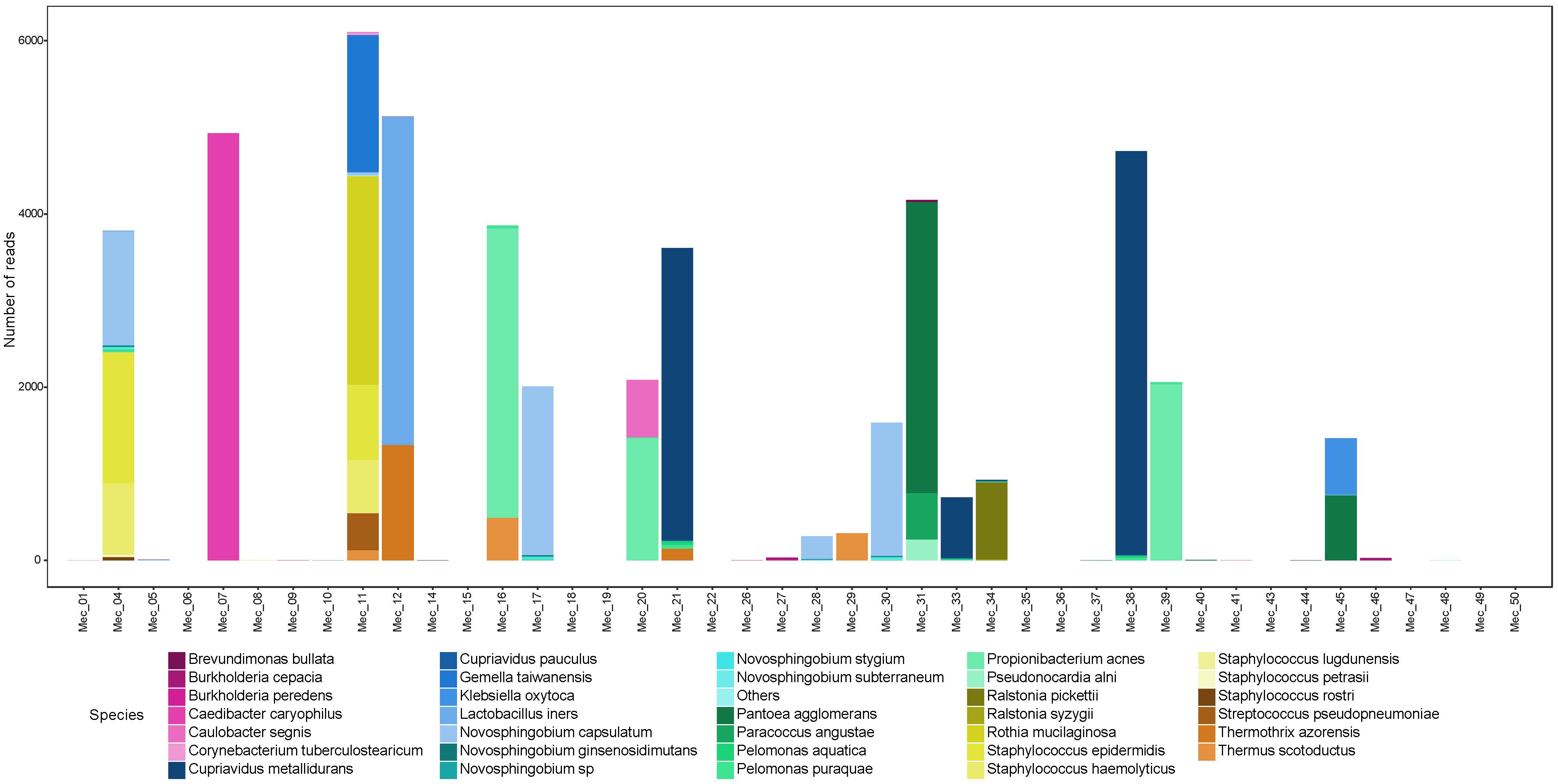

Supplement: Figure S1 — Abundance (number of reads) of bacterial species detected in meconium samples presented with the two dominant P. puraquae OTUs removed. [file Image_1.JPEG]

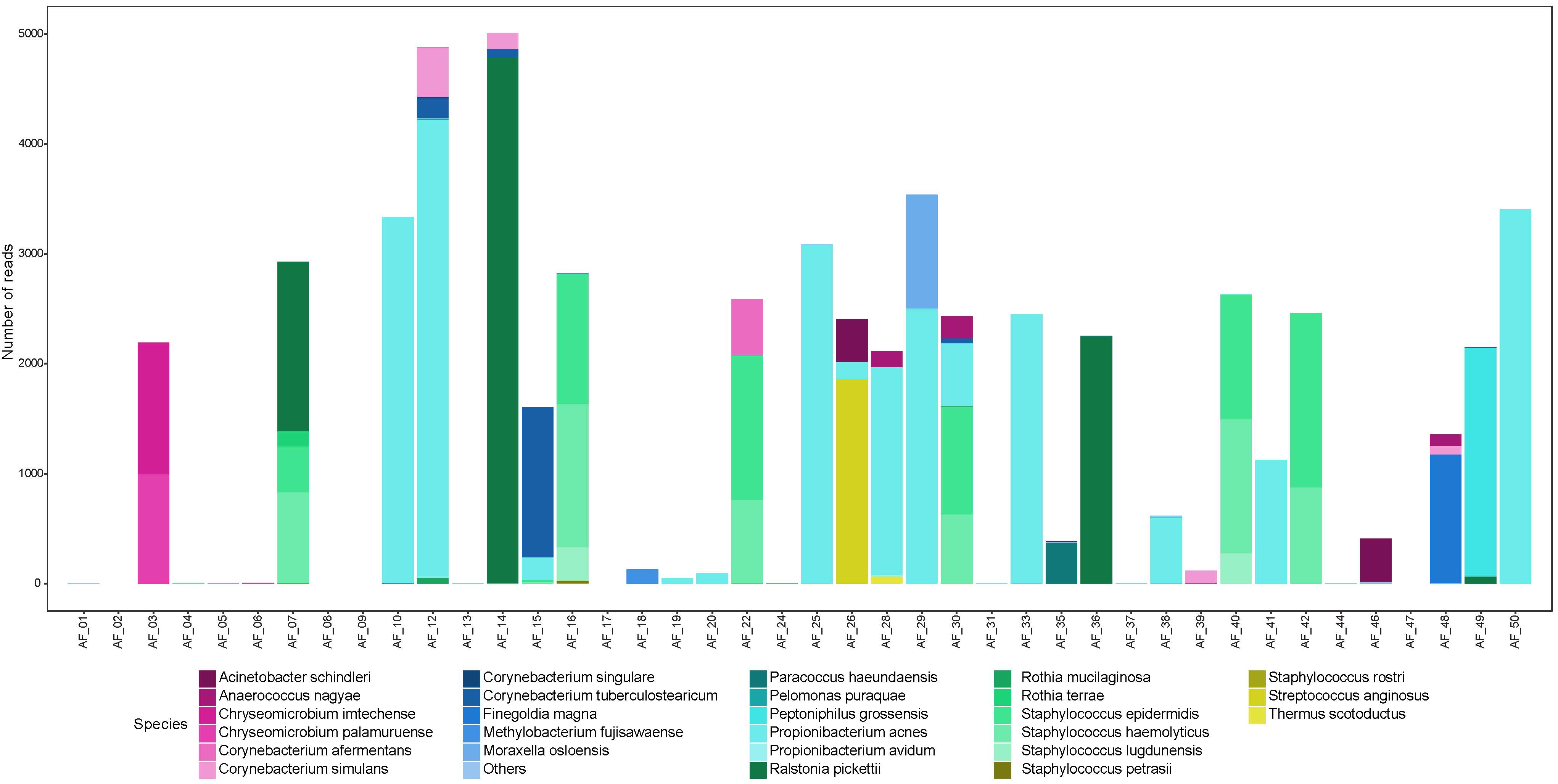

Supplement: Figure S2 — Abundance (number of reads) of bacterial species detected in amniotic fluid samples presented with the two dominant P. puraquae OTUs removed. [file Image_2.JPEG]
